# A Web-Based Patient Empowerment to Medication Adherence Program for Patients With Rheumatoid Arthritis: Feasibility Randomized Controlled Trial

**DOI:** 10.2196/48079

**Published:** 2023-11-06

**Authors:** Siriwan Lim, Ponrathi Athilingam, Manjari Lahiri, Peter Pak Moon Cheung, Hong-Gu He, Violeta Lopez

**Affiliations:** 1 Alice Lee Centre for Nursing Studies Yong Loo Lin School of Medicine National University of Singapore Singapore Singapore; 2 College of Nursing University of South Florida Tampa, FL United States; 3 Division of Rheumatology Department of Medicine National University Hospital Singapore Singapore; 4 Department of Medicine Yong Loo Lin School of Medicine National University of Singapore Singapore Singapore; 5 School of Nursing, Midwifery and Social Sciences Central Queensland University Rockhampton, Queensland Australia

**Keywords:** patient empowerment, rheumatoid arthritis, intervention, feasibility study, adherence, chronic, arthritis, rheumatism, medication, prescription, drug, mapping, design, development, web based, internet based, web-based tool

## Abstract

**Background:**

Living with a chronic illness such as rheumatoid arthritis (RA) requires medications and therapies, as well as long-term follow-up with multidisciplinary clinical teams. Patient involvement in the shared decision-making process on medication regimens is an important element in promoting medication adherence. Literature review and needs assessment showed the viability of technology-based interventions to equip patients with knowledge about chronic illness and competencies to improve their adherence to medications. Thus, a web-based intervention was developed to empower patients living with RA to adhere to their disease-modifying antirheumatic drugs (DMARDs) medication regimen.

**Objective:**

This study aims to discuss the intervention mapping process in the design of a web-based intervention that supports patient empowerment to medication adherence and to evaluate its feasibility among patients living with RA.

**Methods:**

The theory-based Patient Empowerment to Medication Adherence Programme (PE2MAP) for patients with RA was built upon the Zimmerman Psychological Empowerment framework, a web-based program launched through the Udemy website. PE2MAP was developed using a 6-step intervention mapping process: (1) needs assessment, (2) program objectives, (3) conceptual framework to guide the intervention, (4) program plan, (5) adoption, and (6) evaluation involving multidisciplinary health care professionals (HCPs) and a multimedia team. PE2MAP is designed as a 4-week web-based intervention program with a complementary RA handbook. A feasibility randomized controlled trial was completed on 30 participants from the intervention group who are actively taking DMARD medication for RA to test the acceptability and feasibility of the PE2MAP.

**Results:**

The mean age and disease duration of the 30 participants were 52.63 and 8.50 years, respectively. The feasibility data showed 87% (n=26) completed the 4-week web-based PE2MAP intervention, 57% (n=17) completed all 100% of the contents, and 27% (n=8) completed 96% to 74% of the contents, indicating the overall feasibility of the intervention. As a whole, 96% (n=24) of the participants found the information on managing the side effects of medications, keeping fit, managing flare-ups, and monitoring joint swelling/pain/stiffness as the most useful contents of the intervention. In addition, 88% (n=23) and 92% (n=24) agreed that the intervention improved their adherence to medications and management of their side effects, including confidence in communicating with their health care team, respectively. The dos and do nots of traditional Chinese medicine were found by 96% (n=25) to be useful. Goal setting was rated as the least useful skill by 6 (23.1%) of the participants.

**Conclusions:**

The web-based PE2MAP intervention was found to be acceptable, feasible, and effective as a web-based tool to empower patients with RA to manage and adhere to their DMARD medications. Further well-designed randomized controlled trials are warranted to explore the effectiveness of this intervention in the management of patients with RA.

## Introduction

Living with a chronic medical condition renders an individual more vulnerable to the challenges of everyday living. Evidence supports that patients who feel empowered and are actively involved in their care plans have improved adherence to medications and better control of their long-term health condition [1]. Patients living with a chronic illness require medications and therapies, as well as long-term follow-ups with multidisciplinary clinical teams. Rheumatoid arthritis (RA) is a chronic inflammatory arthritis disease with patients experiencing joint pains, fatigue, and physical disability requiring regular long-term medications, follow-ups, and multidisciplinary care. European League Against Rheumatism (EULAR) advocates early diagnosis and the use of disease-modifying antirheumatic drugs (DMARDs) to prevent the destruction of joints and preserve their function, achieve early remission (low disease activity), and improve quality of life in patients with RA [2]. One of the overarching principles of “treat to target (T2T)” in RA management includes shared decision-making between the patient and rheumatologist [2,3]. Traditionally, there has been a lack of coordination among health care professionals (HCPs), and patient education is sporadic and unplanned for patients with RA [4]. Medication adherence among patients with RA has been reported to be suboptimal [5-7]. The involvement of patients with RA in the shared decision-making process on their treatment is an important element in promoting medication adherence [8].

Patients’ reluctance to adhere to RA medications is influenced by low motivation, personal beliefs, advanced age, poverty, limited knowledge of health, and the inability to cope with high drug costs [8]. A systematic review identified cost barriers as the principal factor for nonadherence among patients with RA [9]. Nonadherence to DMARDs was associated with a higher disease activity that increased visits to the emergency department, hospitalization, and the overall cost of care [10,11]. The World Health Organization (WHO) identified these social, patient, disease, treatment, and health care services and patient-HCP relationship-related factors as the 5 dimensions of medication adherence [12]. Given the multiple negative implications of nonadherence, effective interventions that address the nonadherence factors to medication adherence among patients living with RA are needed [7]. Currently, there is a paucity of effective interventions to address nonadherence in patients with inflammatory arthritis [13]. Interventions should target health behavioral changes, including improving patients’ knowledge, motivation, and self-efficacy to manage their condition and long-term adherence to medication. Self-efficacy is related to the competency component of patient empowerment and also the cornerstone of medication adherence among patients with chronic illnesses such as RA [14], who need to manage their symptoms and adjustments of their medication regime through different stages of their illness trajectories [15,16].

A community-based chronic care model has been shown to help patients with RA develop independence and competency in keeping their symptoms under control and maintaining normalization of their social and work lives [17]. The community-based chronic illness model-of-care concept aligns well with promoting patient empowerment and a right for patients to make their own health-related decisions. Empowerment is defined as a helping process, a partnership that values self and others, and mutual decision-making using resources to solve their problems and mobilize the necessary resources to take care of their own lives [18]. Ethically, patient empowerment supports patients’ self-determination and autonomy and is embedded within the patient-centered care frameworks [19]. Therefore, we proposed the development of an empowerment intervention to support patients with RA in managing their medication adherence. This study describes the process involved in the design and development of a web-based empowerment intervention and tests the acceptability and feasibility of the intervention among patients with RA.

## Methods

### Overview

The principal investigator (PI) followed a 6-step intervention mapping process, including needs assessment, developing program objectives, a conceptual framework for the development of the intervention, program plan, adoption, and evaluation [20]. This process has been implemented successfully in the development of a mobile health intervention for self-care of patients with heart failure [21], breast and cervical cancer screening [22], and web-based, tailored intervention that promotes vaccination acceptance [23].

### Step 1: Needs Assessment

The main author (SL), who is also the PI, conducted a literature review to critically explore, evaluate, and synthesize the findings of selected studies on patient empowerment and interventions that promote empowerment and medication adherence among people living with chronic illnesses such as RA. The findings from the literature review informed the authors on the concept of patient empowerment and how that influenced the patient’s adherence to medications. It also guided the adoption of an appropriate empowerment framework, which will be described in step 3.

The findings from the literature review were used to develop a semistructured question interview guide to understand the perception of empowerment and medication adherence among patients with RA and their HCPs in our local context.

A qualitative interview study was carried out on 28 patients with RA and 17 HCPs from a local health care institution using the semistructured guide. The data identified five factors that hindered empowerment and medication adherence by patients with RA and HCPs: (1) limited consultation period with the doctors, (2) limited health literacy, (3) burden of medical cost, (4) lack of peer-sharing, and (5) lack of awareness and access to resources. The data also revealed four themes that supported empowerment and medication adherence: (1) accepting and coming to terms with RA, (2) taking control of own health, (3) harnessing support from others, and (4) adhering to medications. These findings were incorporated into the “Patient Empowerment to Medication Adherence Programme” (PE2MAP) intervention through reinforcement of their health literacy, self-care competency, and family support.

### Step 2: Program Objectives

The primary objective was to design a web-based intervention that supports patient empowerment to medication adherence. The secondary objective was to pilot-test the feasibility of the web-based intervention among patients with RA.

### Step 3: Conceptual Framework

**Figure 1:**
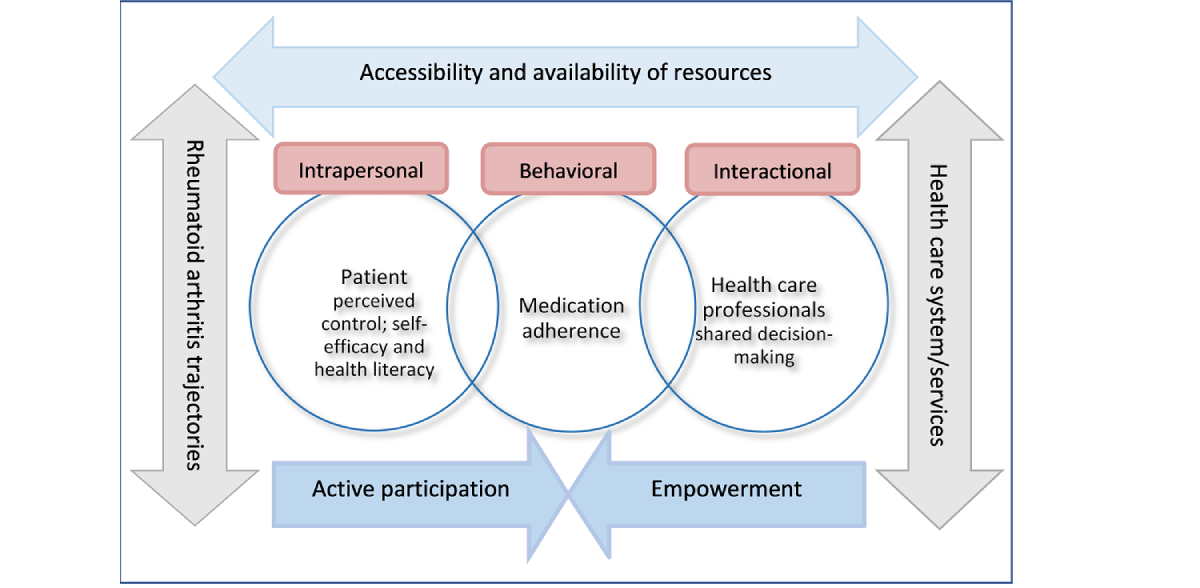
Psychological empowerment framework for developing patient empowerment to medication adherence programme (PE2MAP).

The theoretical framework used in the development of PE2MAP, a 4-week web-based intervention with a complementary RA handbook, was built upon psychological empowerment (PE) [24]. The PE framework explores 3 levels of empowerment (ie, intrapersonal, interactional, and behavioral levels) to maximize RA patients’ health potential and their adherence to medication (Figure 1).

The intrapersonal level addresses perceived competency, control, and self-efficacy, while the interactional level addresses resource mobilization. Patients need resources such as information about RA (knowledge), DMARDs, and problem-solving skills that may help them adapt, control, and cope with their distressing symptoms such as joint pain, stiffness, and physical limitations. Understanding the actions, benefits, and potential side effects of DMARDs may seem complicated for patients. Therefore, it requires time and a need for a close partnership with their HCPs to maintain adherence to their treatment. The behavioral level addresses patients’ participation in decision-making, medication adherence, and coping ability. Patients’ active participation in their treatment is paramount to working through the challenges in managing their RA trajectories with reviews and adjustments required of their medication regimens [15,25].

### Step 4: Program Plan

The PE2MAP intervention program was developed based on gaps identified in the literature review and the findings from the need assessment at the intrapersonal, interactional, and behavioral levels. The literature and the needs assessment showed the viability of technology-based interventions to enhance patients’ control over their pace of learning, medication adherence, and tracking of illness trajectory through self-monitoring of pain, and fatigue levels.

The PI worked with a team comprising of rheumatologists, nurses, and allied health from the One-Stop Arthritis Clinic (OSAC) to develop the PE2MAP’s educational video storyboards that included the RA disease process, types of DMARDs, and self-care monitoring and self-management. The PI also worked in consultation with a team of web designers to develop PE2MAP as a technology-based web-based intervention. The contents of PE2MAP were presented using animation and in-house videos. The disease process of RA, actions of DMARDs, treatments, and commonly prescribed tests and investigations were explained using animation in a visually graphical way that was easy for the layperson to understand. The voiceover for the animation videos in PE2MAP was recorded by a professional voice artist.

Peer-sharing videos by 2 patients living with RA on how they learned to manage their symptoms, medication, and social activities were also included. All the videos have Mandarin subtitles to cater to older Chinese-speaking patients. The videos were pilot-tested with 2 patients with RA and 2 HCPs to gather their feedback for improvement.

Table 1 below describes the 4 animation videos, 12 competency-building videos, 2 peer-sharing videos, 4 quizzes to reinforce patients’ knowledge, and the topics covered over the 4-weeks in PE2MAP. Once the contents were approved by the team, the team obtained clearance from the institution’s corporate communication department. Finally, the PI worked with the main web designer to interface the contents and layout of the web-based PE2MAP on Udemy.

In addition, the RA handbook contained goal-setting guidelines, pictorials, and tables to guide the patients in identifying their DMARDs and monitoring their pain and fatigue levels, blood results (inflammatory markers), and medication intake. Patients used the RA handbook to complement their PE2MAP web-based learning and record their experiences as they went through the intervention period. The planning and completion of PE2MAP web-based content and the RA handbook took about 6 months.

**Table 1:** Contents included in PE2MAP^a^.

Title of video on each week			Tokens awarded	
**Week 1**				
	Understanding Rheumatoid Arthritis	N/A^b^		
	Multidisciplinary team: Part 1 and Part 2	N/A		
	Complete Quiz 1	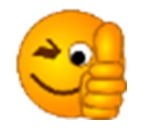		
	Online discussion forum	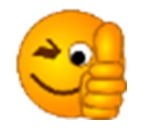		
**Week 2**				
	Understanding RA^c^ medications #DMARDs^d^; #Steroids; #NSAIDs^e^	N/A		
	Communication with health care professional: (Part 1)	N/A		
	Communication with health care professional: role play Patient-Doctor (Part 2)	N/A		
	Complete Quiz 2	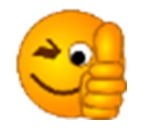		
	Online discussion forum	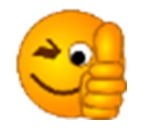		
**Week 3**				
	Communication with health care professional: role play patient-nurse (medication)	N/A		
	Demonstration of mobile application	N/A		
	Communication with health care professional: role play patient-nurse (side effects medication)	N/A		
	Communication with health care professional: role play patient-MSW^f^	N/A		
	Complete Quiz 3	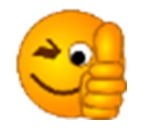		
	Online discussion forum	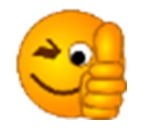		
**Week 4**				
	Managing Foot Pain (podiatrist)	N/A		
	Managing Joint Pain or Stiffness (occupational therapist)	N/A		
	Managing joint pain/stiffness (physiotherapist)	N/A		
	Communication with health care professional: role play Patient-Nurse (quitting smoking)	N/A		
	Complete Quiz 4	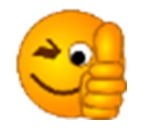		
	Online discussion forum	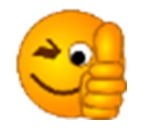		
**Week A**				
	Peer Sharing Part 1 (Pt.^g^ 1)	N/A		
**Week B**				
	Peer Sharing Part 2 (Pt. 2)	N/A		

^a^PE2MAP: Patient Empowerment to Medication Adherence Programme.

^b^N/A: not applicable.

^c^RA: rheumatoid arthritis.

^d^DMARD: disease-modifying antirheumatic drug.

^e^NSAID: nonsteroidal anti-inflammatory drug.

^f^MSW: medical social worker.

^g^Pt.: patient.

### Ethical Considerations

Ethics approval was obtained from the National Health Care Group Domain Specific Review Board (NHG-DSRB; Reference Number: 2016/00500) before data collection. All participants in the study were informed that they could withdraw from the study at any time and the participants’ anonymity in the study was ensured prior to getting informed consent from them. They were also informed that they would receive an honorarium amount of SGD $50 (with an average currency exchange rate of SG $1=US $0.73) for their participation in the study.

### Step 5: Adoption of Web-Based PE2MAP

**Figure 2:**
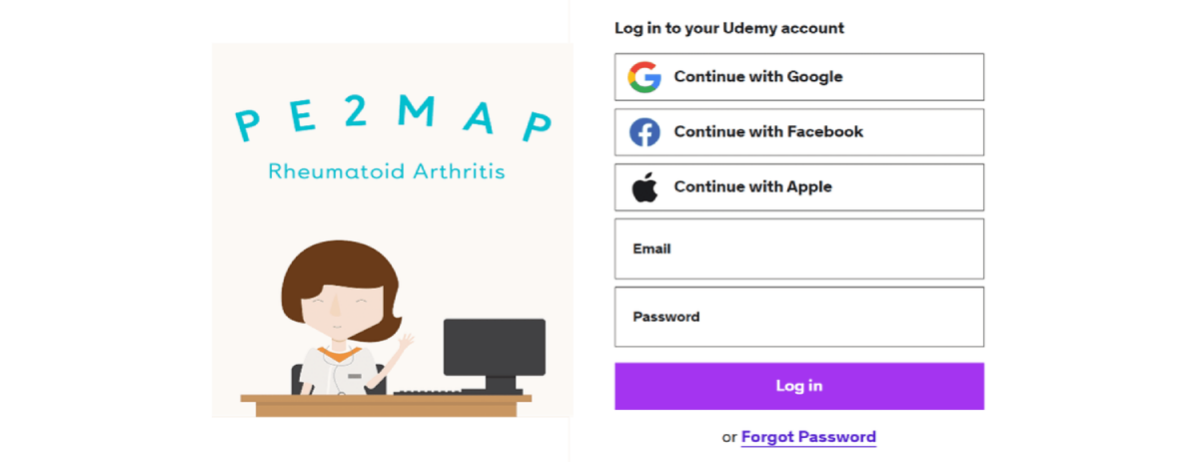
Login page for PE2MAP in Udemy. PE2MAP: patient empowerment to medication adherence programme.

**Figure 3:**
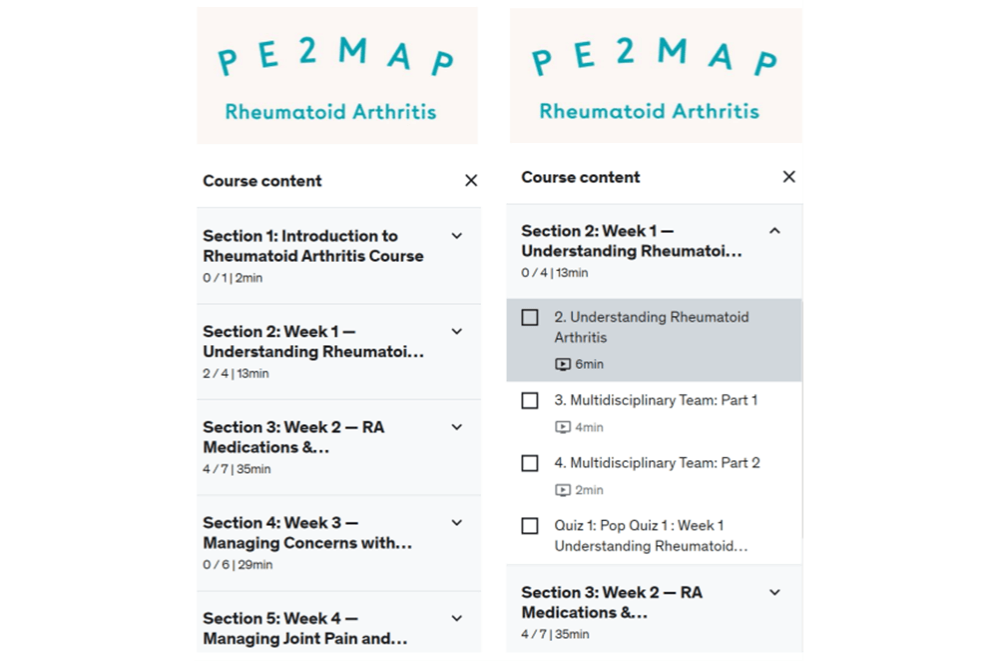
Course content for 4 weeks and detailed content for week 1. PE2MAP: patient empowerment to medication adherence programme.

The PE2MAP was launched through the Udemy website, a web-based education platform. Web-based PE2MAP access was granted through an individual’s email address with password protection for participants enrolled in the study (Figure 2). A detailed description of the topics covered over the 4 weeks can be found in Table 1 and Figure 3. There is no payment involved for the participants to be enrolled and use the PE2MAP intervention. Participants received weekly reminders to view weekly videos and contents of the PE2MAP, and complete the weekly quiz.

Participants were given an instruction handbook to guide them through the steps to log in to PE2MAP on Udemy, which was illustrated using pictures and short descriptions.

### Detail on PE2MAP Intervention

**Figure 4:**
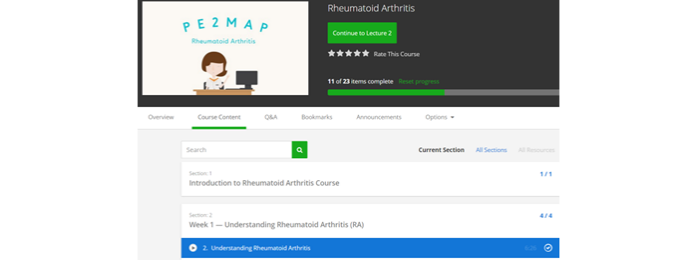
Progression through the weekly PE2MAP intervention. PE2MAP: patient empowerment to medication adherence programme.

Participants received notification from the PI at the beginning of each week about the content of that week’s content to be reviewed. As indicated in Figure 2, participants accessed PE2MAP on the Udemy web page using the individual email and password assigned to them. Once they log in to the PE2MAP home page, the participants are able to browse the weekly content from the drop-down menu, as shown in Figure 3, and complete the weekly quiz. Upon completion of the videos and quiz for the prescribed week, as indicated in Figure 4, the contents completed each week are shown and participants proceed to the following week’s content. Participants who missed the reminder were contacted by the PI and were offered additional support to navigate Udemy’s platform and were encouraged to make time to complete the weekly content.

### Feedback on Quiz Completion

**Figure 5:**
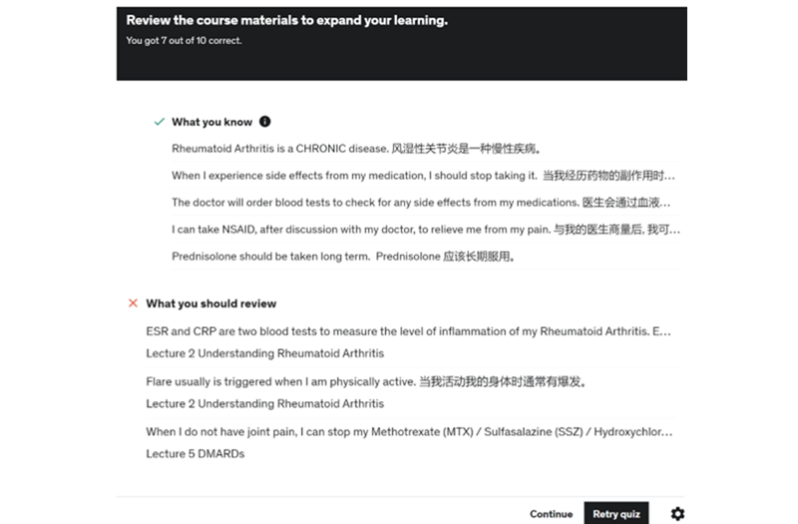
Performance feedback on quiz.

Participants received immediate feedback on their performance on weekly quizzes. As indicated in Figure 5, the feedback included the areas the participants lacked understanding and directed them to review the relevant videos and attempt the quiz again. The PI would also send weekly reminders to prompt participants to log in to their Udemy account to view the week’s videos and complete the quiz.

### Following Up on Participants’ Weekly Progress

**Figure 6:**
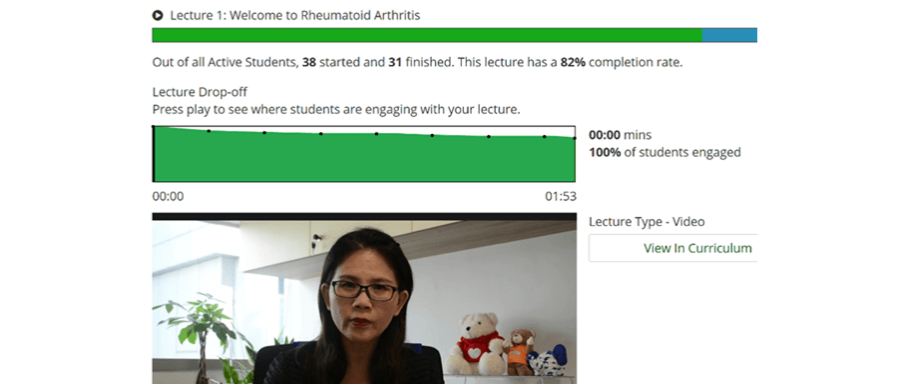
Tracking the progress of participants.

The PI viewed the content developer’s dashboard and data analytics on the Udemy website weekly as seen in Figure 6. The PI would contact the participants who did not complete 100% of the weekly web-based content to understand the rationale and confirm if they have acquired the competencies or knowledge about their condition and DMARDs since these participants were more selective about the content they reviewed in PE2MAP.

### Step 6: Evaluation

The PE2MAP was evaluated for feasibility among 30 patients with RA. Participants were included if they were adults aged 21 years or older with a clinical diagnosis of RA and actively taking medications, and either able to read or speak in English or, if unable to, have a family member who can read English and assist with the translation into the patient’s spoken language.

## Results

### Demographics

As shown in Table 2, the mean age of the 30 participants was 52.63 (SD 14.12) years, and 80% (n=24) were above 40 years of age. About 70% (n=21) were Chinese, 80% (n=24) were females, and 50% (n=15) had completed secondary education. The mean duration of RA was 8.50 (SD 8.55) years and about 47% (n=14) had the disease for less than 5 years, and 33% (n=10) of participants reported having more than 2 chronic disease conditions. The most common DMARDs prescribed for the participants were methotrexate (18/30, 60%), hydroxychloroquine (12/30, 40%), and sulfasalazine (8/30, 26.7%), and some participants were taking multiple drugs.

**Table 2:** Demographics of participants enrolled in the feasibility study (N=30).

Demographics of participants		Values
Age (years), mean (SD)		52.63 (14.12)
**Age (years), n (%)**		
	<40	6 (20)
	40-55	11 (36.7)
	>55	13 (43.3)
**Gender, n (%)**		
	Male	6 (20)
	Female	24 (80)
**Marital status, n (%)**		
	Married	23 (76.7)
	Single and others	7 (23.3)
**Race, n (%)**		
	Chinese	21 (70)
	Malay or Indian or others	9 (30)
**Religion, n (%)**		
	Buddhism	7 (23.3)
	Christianity	7 (23.3)
	Muslim	4 (13.3)
	Hindu	3 (10)
	Freethinkers and others	9 (30)
**Education, n (%)**		
	Primary and secondary	15 (50)
	Diploma or technical	6 (20)
	Degree above	9 (30)
**Monthly personal income (SGD)^a^, n (%)**		
	<SG $2500	15 (50)
	SG $2501-SG $4500	7 (23.3)
	>SG $4501	8 (26.7)
**RA^b^ disease duration range, n (%)**		
	0-5	14 (46.7)
	6-10	8 (26.7)
	>10	8 (26.7)
**Number of DMARDs^c^, n (%)**		
	1	18 (60)
	2	10 (33.3)
	3	2 (6.7)
**Types of DMARDs, n (%)**		
	Methotrexate	18 (60)
	Hydroxychloroquine	12 (40)
	Sulfasalazine	8 (26.7)
	Leflunomide (Arava)	6 (20)
**Other medications** **, n (%)**		
	NSAID^d^	7 (23.3)
	Prednisolone	19 (63.3)

^a^SGD: Singapore Dollar (with an average currency exchange rate of SG $1=US $0.73).

^b^RA: rheumatoid arthritis.

^c^DMARD: disease-modifying antirheumatic drug.

^d^NSAID: nonsteroidal anti-inflammatory drug.

### PE2MAP Intervention Completion Rate

**Figure 7:**
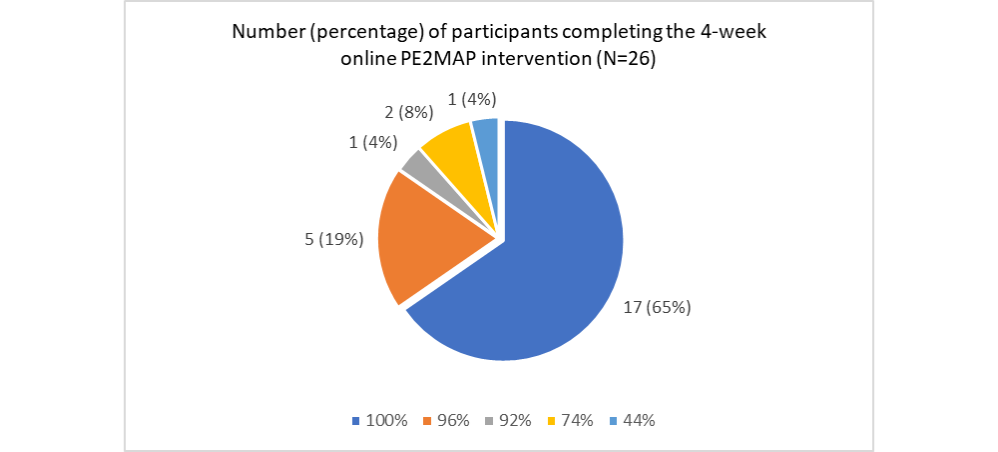
PE2MAP percentage of intervention content completion. PE2MAP: patient empowerment to medication adherence programme.

As shown in Figure 7, of the 30 participants enrolled, 87% (n=26) completed the 4-week web-based PE2MAP intervention. Among the 26 participants that completed the 4-week intervention, the percentages of completion of the web-based content were 100% (n=17), 96% (n=5), 92% (n=1), 74% (n=2), and 44% (n=1).

Among the 4 participants who did not complete the study within the 4-week intervention period, most reported conflict with their work schedule (n=3), and 1 participant, a retiree, lacked knowledge on the use of the internet and did not want to bother family members in using PE2MAP.

### Evaluation of the Web-Based PE2MAP on Usability

Table 3 below shows the benefits of the contents of the web-based PE2MAP as indicated by the participants. All 100% (n=26) of the participants reported (agree or strongly agreed) that they gained more knowledge, and 96% (n=25) reported that they gained new skills and benefitted from participating in the PE2MAP intervention. About 88% (n=23) and 92% (n=24) agreed that the intervention improved their adherence to medications and management of the side effects, respectively. At the same time, 92% (n=24) gained confidence in communication skills to engage with their health care team and 96% (n=25) unanimously reported that they would recommend PE2MAP to other patients with RA.

**Table 3:** Evaluation of participants’ reported benefits from PE2MAP^a^ (N=26; one participant did not answer Q2 to Q5).

Feature of PE2MAP^a^	Strongly disagree, n (%)	Disagree, n (%)	Agree, n (%)	Strongly agree, n (%)
1. I have gained more knowledge about RA^b^	0 (0)	0 (0)	13 (50)	13 (50)
2. I have learned to adhere to my medication regime	0 (0)	2 (7.7)	13 (50)	10 (38.4)
3. I know how to manage the side effects of my RA medications	0 (0)	1 (3.8)	17 (65.4)	7 (26.9)
4. I know how to communicate better with my doctor and other health care professionals during consultation	0 (0)	1 (3.8)	14 (53.9)	10 (38.4)
5. I have learned new skills to better manage my RA	0 (0)	0 (0)	16 (61.5)	9 (34.6)
6. Overall, I have benefitted from PE2MAP	0 (0)	0 (0)	13 (50)	12 (46.2)

^a^PE2MAP: Patient Empowerment to Medication Adherence Programme.

^b^RA: rheumatoid arthritis.

### Evaluation of RA Handbook

As included in Table 4 below, overall, 96% (n=25) of the participants found the information on managing the side effects of medications, 96% (n=25) on keeping fit, 96% (n=25) on managing flare-ups, 96% (n=25) on monitoring joint swelling or pain or stiffness, and 96% (n=25) on monitoring blood results, as the most useful information in the handbook. In addition, the dos and do nots of traditional Chinese medicine were found by 96% (n=25) to be useful. Goal setting was rated as the least useful skill by 6 (23.1%) of the participants.

**Table 4:** Evaluation of the features of RA^a^ handbook (N=26; one participant omitted Q1 and Q12).

Features of RA handbook	Least useful, n (%)	Somewhat useful, n (%)	Most useful, n (%)
1. Goal setting (n=25)	6 (23.1)	12 (46.1)	7 (26.9)
2. Medications (n=26)	1 (3.8)	14 (53.8)	11 (42.3)
3. Managing the side effects of medications (n=26)	1 (3.8)	13 (50)	12 (46.1)
4. Monitoring of pain score (n=26)	1 (3.8)	16 (61.5)	9 (34.6)
5. Monitoring joint swelling or pain or stiffness (n=26)	1 (3.8)	13 (50)	12 (46.1)
6. Managing flare (being aware of triggers, managing emotions, and getting enough sleep; n=26)	1 (3.8)	12 (46.1)	13 (50)
7. Managing fatigue (n=26)	1 (3.8)	14 (53.8)	11 (42.3)
8. Dos and do nots about the use of TCM^b^ (n=26)	1 (3.8)	13 (50)	12 (46.1)
9. Monitoring blood results (n=26)	1 (3.8)	8 (30.8)	17 (65.4)
10. Keeping fit (n=26)	1 (3.8)	11 (42.3)	14 (53.8)
11. Diet (n=26)	0 (0)	15 (57.7)	11 (42.3)
12. Support (n=25)	0 (0)	12 (46.1)	13 (50)

^a^RA: rheumatoid arthritis.

^b^TCM: traditional Chinese medicine.

## Discussion

### Principal Findings

According to the literature, adherence to medication in patients with RA is low, varying from 30% to 80%. Medication adherence is a multidimensional concept and requires a multidisciplinary approach, including all stakeholders to make informed decisions about their treatment plan. Thus, the primary objective of developing the web-based PE2MAP intervention was to empower patients with RA to improve their medication adherence. We worked closely with a multidisciplinary team and followed a 6-step intervention mapping process to methodically design and test the PE2MAP for feasibility among the end users who are patients with RA. Each step of the intervention mapping process was followed carefully and explained in the Results section. The intervention and outcome measurements were aligned with the intrapersonal, interactional, and behavioral levels of the psychological empowerment (PE) framework. The contents included in PE2MAP were verified by clinical experts and patients. Currently, there are limited studies that have an underpinning theoretical framework and report user engagement with web-based interventions among patients with inflammatory arthritis [26].

Our study team recruited a total of 30 participants, and 86% (n=26) of the patients completed the 4-week intervention. The reasons for dropout were conflicts with their other commitments and lack of confidence accessing the internet. Of the 26 participants who completed the 4-week intervention, the percentage of completion of the web-based content was 100% by 17 participants and 96% by another 5 participants, indicating a high completion of the 4-week intervention by almost 84% (n=22) of the participants. Results from a randomized controlled trial study of 74 patients with RA reported a 78% mean attendance rate in the intervention group for physical activity [27]. Our result is encouraging, considering that most of the participants are 40 years or older, with secondary education and established RA, indicating that the PE2MAP is feasible for use among patients with RA.

Most studies report the final study completion rate rather than the intervention completion rate or intervention dose. We have included the percentage of intervention dose completion. These results are supported by a family empowerment study of 60 participants who reported a mean intervention completion rate of 77% among the intervention group [28]. On the other hand, the intervention completion rate was reported as low as 30.6% in a psychosocial intervention study of the effectiveness of substance abuse [29]. An 80% completion rate was reported by 2 feasibility studies of the mobile app for heart failure self-management [30,31], supporting our study results. Therefore, we are confident that patients with RA would benefit from the PE2MAP intervention.

The usefulness and benefits of the contents included in PE2MAP were reported as favorable in that 92% (n=24) of the participants gained more knowledge and self-efficacy in communicating with their HCPs and managing their medication regime.

Research has shown that simply providing patients with information or advice was not sufficient to change health behaviors or improve knowledge [32]. A meta-analysis showed that the use of mobile phone messaging apps may provide benefits in supporting the self-management of long-term illnesses in patients with diabetes, asthma, hypertension, and others [33]. This supports the development of PE2MAP intervention to be appropriate and engaging.

### Strengths and Limitations

The major strength of the web-based PE2MAP is the use of the intervention mapping process and the empowerment framework, addressing gaps identified in the design and testing of the intervention to improve medication adherence among patients with RA. The feasibility testing among a small number of patients with RA is encouraging with 88% (n=23) of the participants completing 92% to 100% of the intervention, indicating that the PE2MAP is acceptable and feasible among patients with RA. The RA handbook and web-based PE2MAP complement one another to empower the participants to build their knowledge and skills competency and manage their RA condition and adhere to their medications.

There were several limitations in this study. First, the PE2MAP was a web-based program that required the participants to have access to the internet. Participants were unable to log in to PE2MAP when there was no internet connection, with concerns about incurring extra costs on a mobile data plan if they could only access data from their smartphones. Second, participants in the testing of the acceptability and feasibility of PE2MAP include a small sample of only 30 participants who were mostly married, retired, and Chinese women. Last, this study lacked formal goal setting and included it as optional in the handbook. Thus goal setting was considered least useful by 23% (n=6) of participants.

### Conclusions

Our current prototype of web-based PE2MAP intervention for patients with RA demonstrated acceptability and feasibility among the participants in this study. The use of animation and competency-building videos, peer sharing, and weekly quizzes scaffolded over 4 weeks including a complementary RA handbook, and weekly reminder and follow-up by the PI address the intrapersonal, interactional, and behavioral levels of the PE framework. The favorable completion rate and evaluation of the contents showed that PE2MAP improves the participants’ knowledge and competencies needed to self-manage their illness and self-efficacy to adhere to their DMARDs medication regime.

However, further refinements to the program are required by including goal-setting components such as health coaching within the program, not in the RA handbook. Future studies could recruit participants from different types of inflammatory arthritis and socioeconomic backgrounds. A full-scale randomized controlled trial is warranted to test the effectiveness of the updated PE2MAP intervention besides a web-based platform such as a mobile app before implementing the intervention as a standard of care for patients with RA.

## Appendix

Multimedia Appendix 1 CONSORT E-Health.

## Abbreviations

DMARD: disease-modifying antirheumatic drug
HCP: health care professional
PE: psychological empowerment
PE2MAP: Patient Empowerment to Medication Adherence Programme
PI: principal investigator
RA: rheumatoid arthritis

## Data Availability

All data generated or analyzed during this study are included in this published paper.
